# Intratumoral microbiome and gastrointestinal cancers

**DOI:** 10.3389/fonc.2022.1047015

**Published:** 2022-11-29

**Authors:** Shengnan Li, Qian Li, Wei Lu

**Affiliations:** ^1^ Department of Hepatology, Tianjin Second People’s Hospital, Tianjin Institute of Hepatology, Tianjin, China; ^2^ Department of Hepatobiliary Oncology, Liver Cancer Center, Tianjin Medical University Cancer Institute and Hospital, National Clinical Research Center for Cancer, Key Laboratory of Cancer Prevention and Therapy, Tianjin’s Clinical Research Center for Cancer, Tianjin Medical University, Tianjin, China

**Keywords:** tumor microenvironment, intratumoral microbiome, gastrointestinal cancer, commensal microbiota, tumor metabolism

## Abstract

Emerging studies have revealed the role of microbiota in regulating tumorigenesis, development, and response to antitumor treatment. However, most studies have focused on gut microbiota, and little is known about the intratumoral microbiome. To date, the latest research has indicated that the intratumoral microbiome is a key component of the tumor microenvironment (TME), and can promote a heterogeneous immune microenvironment, reprogram tumor metabolism to affect tumor invasion and metastasis. In this review, we will summarize existing studies on the intratumoral microbiome of gastrointestinal cancers and reveal their crosstalk. This will provide a better understanding of this emerging field and help to explore new therapeutic approaches for cancer patients by targeting the intratumoral microbiome.

## Introduction

Interactions between the microbiome and human body are well known to be complex (1, 2). The microbiome can affect different physical processes in several ways, most importantly through metabolism and immunity. Approximately 20% of human malignant tumors are associated with the microbiome (3), of which gastrointestinal cancers, including *Helicobacter pylori*-associated gastric cancer, hepatitis B virus-associated hepatocellular carcinoma (HCC), and *Fusobacterium nucleatum*-associated colorectal cancer (CRC), account for a vast proportion.

The commensal microbiota mainly resides in the gut (4). Emerging evidence has indicated an association between gut dysbiosis and various tumors (5), as well as revealed the potential of gut microbiota as a non-invasive diagnostic marker for tumors. For example, Ren et al. reported a decrease of butyrate-producing bacteria but increased lipopolysaccharide-producing bacteria in early HCC (6). Similarly, a decrease of methanogenic archaea, *Saccharomycetes*, and *Pneumocystidomycetes*, but enrichment of halophilic archaea, *Malasseziomycetes* in fecal samples of patients with CRC were also found (7). In addition, a series of microbial prognostic models are established to discriminate cases from control individuals (6, 8). Moreover, the dysbiosis of microbiome is stage-specific and specific microbial markers are associated with the survival of patients, independently of tumor stage, lymph node metastases, or clinical parameters (8). Gut dysbiosis can lead to alterations in key proteases and metabolites, such as toll-like receptor, nuclear factor-kappa B (NF-κB), and short-chain fatty acids, regulate immunity and metabolism so as to induce tumorigenesis and development (9). Furthermore, the key role of gut microbiota in mediating tumor responses to chemotherapy and immunotherapy has also been highlighted (10, 11).

Considering the outstanding progression of gut microbiota, studies on intratumoral microbiome have also substantially advanced in recent years. The intratumoral microbiome is reported to interact with TME and play important roles in regulating tumorigenesis, development, and response to antitumor treatments (1, 12, 13). Here, we will review studies on the intratumoral microbiome of gastrointestinal cancers.

## Intratumoral microbiome of gastrointestinal cancer

As early as in the last century, scientists have detected the presence of bacteria in tumor tissues (14). However, characterizing the intratumoral microbiome remains difficult because of the extremely low microbial biomass of tumors and limited detection technology. With the development of next-generation sequencing technology, emerging studies have begun to explore the composition of the intratumoral microbiome and its role in tumorigenesis and progression. A recent study detected intratumoral bacteria in 1526 tumor tissues of melanoma, pancreatic cancer, lung cancer, ovarian cancer, glioblastoma, bone cancer, and breast cancer using multiple technologies (e.g., 16S rRNA sequencing, immunohistochemistry, immunofluorescence hybridization, and bacterial culture) (15). Intratumoral bacteria were found to be tumor-specific and associated with smoking history and immunotherapy response. Moreover, they may affect tumor occurrence, development and their therapeutic responses by regulating inflammation and immunity, participating in metabolic processes, and destroying DNA stability (16). In the next section, we will elaborate on the research progress about intratumoral microbiome in different types of gastrointestinal cancers.

### Esophageal cancer

Esophageal cancer is the fourth leading cause of cancer-related death in China with 5-year survival rate less than 20%. It is associated with squamous dysplasia, alcohol consumption, cigarette smoking, and dietary habits (17, 18). Epidemiologic studies have shown the association between variations of the esophageal microbiota and esophageal disease (19, 20). For example, normal esophagus was found to be with higher abundance of *Streptococcus*, while esophagitis and Barrett’s esophagus is enriched in gram-negative bacteria, such as *Veillonella*, *Prevotella*, *Haemophilus*, *Neisseria*, *Granulicatella*, and *Fusobacterium* (21). In contrast, esophageal cancer is associated with specific Gram-negative bacteria (e.g., *Escherichia coli* and *Fusobacterium nucleatum*) (22, 23). Similarly, a decrease of *Veillonella* and *Granulicatella* while an enrichment of *Lactobacillus fermentum* were found in esophageal adenocarcinoma (EAC) patients compared to controls and Barrett’s esophagus patients (24, 25). Attentionally, the relative abundance of *Fusobacterium* spp. was also gradually increased from physiological normal esophagus to esophageal squamous cell carcinoma (ESCC) (26), associated with advanced tumor stage and poor overall survival (27), while the abundance of Proteobacteria was decreased. In a word, esophageal cancers of various pathological types are all complicated with microbial dysbiosis, meanwhile, with decreased microbial diversity compared with control individuals (24, 26).

Although the composition and diversity of the esophageal microbiota correlate with esophageal disease (22), while most data on esophageal microbiota are derived from small-scale cross-sectional studies and the evidence is insufficient to assume causality.

### Gastric cancer

Gastric cancer is a common gastrointestinal cancer and a leading cause of cancer-related death. Proteobacteria (e.g., *H. pylori*) are primarily detected in gastric cancer (28, 29) and have been reported to promote precancerous lesions, such as gastric atrophy, intestinal metaplasia, and atypical hyperplasia, which can eventually lead to gastric cancer (30). Previous hypotheses suggest that an acidic microenvironment in the stomach causes a lack of bacterial diversity. While the microbial diversity in gastric cancer have been found significantly increased as compared with that in chronic gastritis and intestinal metaplasia, with higher relative abundance of *Streptococcus* (31, 32). Microbial alpha-diversity increases with disease severity, that is, chronic gastritis has the lowest microbial diversity, whereas gastric cancer has the highest (31). As reported, *Proteobacteria*, *Firmicutes*, *Bacteroidetes*, *Fusobacteria*, and *Actinobacteria* are the dominant bacteria at the phylum level, and potential cancer-promoting bacteria (e.g., *Lactobacillus*, *Escherichia-Shigella*, *Lachnospiraceae*) are enriched in gastric cancer at the genus level (33). This is consistent with the discovery in other types of tumors (34–36). Altered microbiota in gastric cancer (e.g., bacterial overgrowth and diversified microbial community) might potentially promote inflammation and carcinogenesis (33).

The community structure and microbial diversity of gastric cancer remain poorly understood. Nonetheless, bacterial overgrowth is potentially associated with the development of gastric cancer, and the microbial community construct in gastric cancer and its potential role in carcinogenesis also remain to be further explored.

### Pancreatic cancer

Pancreatic cancer is one of the most aggressive human malignancies with a five-year survival rate of 8%. The pancreas used to be considered a sterile organ. However, emerging studies have demonstrated the presence of bacterial species in the pancreas. A variety of bacteria were detected in 76% of pancreatic ductal adenocarcinoma (PDAC) tissues with a higher proportion in pancreatic cancer tissues compared with normal pancreatic tissues (12, 37). *Proteobacteria*, *Bacteroidetes, and Firmicutes* were found to be the dominant phyla in tumor tissues (12). Higher alpha-diversity of tumor microbiome and high abundances of a microbial signature (*Pseudoxanthomonas-Streptomyces-Saccharopolyspora-Bacillus clausii*) were correlated with longer survival, which could contribute to the anti-tumor immune response by favoring recruitment and activation of CD8+ T cells (11, 38). In addition, bacterial species cultured from fresh PDAC tumor tissues were found to induce resistance to chemotherapeutic drug gemcitabine by preclinical study (37). Beyond intratumoral bacteria, Aykut et al. explored the fungal community of PDAC (39, 40). They showed that fungi migrated from gut lumen to the pancreas, and PDAC showed an alarming increase in fungi compared to normal pancreatic tissue both in humans and mouse models. Specifically, patients with PDAC could be distinguished from healthy individuals by the abundance of markedly enriched *Malassezia* spp.

Attentionally, the origin of tumor-associated bacteria is also a research hotspot. Considering constant interactions between the pancreas and gut, the development of pancreatic cancer is closely related to the dysregulation and mislocation of gut microbiota (12, 41). Consistent with the above, studies have estimated that PDAC-associated bacteria can be translocated from the gastrointestinal tract in a retrograde manner (12, 37). These findings provide the possibility for further exploration of intratumoral microbiome and for development of new strategies for the diagnosis and treatment of pancreatic cancer (42).

### CRC

CRC is a common malignant tumor of the digestive tract with poor prognosis (43). The etiology of CRC involves genetic and environmental factors (44), in which dietary habits, obesity, and heavy drinking play important roles in the occurrence of sporadic CRC. Furthermore, emerging studies have shown the potential role of gut microbiota in the development, diagnosis and treatment of CRC (7, 45–49).

Compared with control individuals, there is no doubt that the gut microbiota in CRC patients is dysbiosis (50–53). Gut commensal bacteria (e.g., *E. coli*, *Fusobacteria*, enterotoxin-producing *Bacteroides fragilis*, and *Peptostreptococcus anaerobius*) have been found to be increased in patients with CRC than in healthy individuals (54–60). *Enterococcus faecalis*, *Salmonella* (61) and *F. nucleatum* are also revealed with significant associations with CRC (56, 62, 63). Special species have been shown to promote tumor cell proliferation *in vitro* and *in vivo* (64), and even predict a poor prognosis (51, 65). Although studies on fungal communities of CRC are limited, but some new findings have been obtained. Coker et al. revealed higher *Basidiomycota: Ascomycota* ratio in CRC patients and different clusters of fungal components in early-stage and late-stage CRC patients, indicating that mycobiome profiles were stage-specific (7). *Ascomycota*, *Glomeromycota*, and *Basidiomycota* were found to be the dominant phyla by characterized fungal microbiota profiles in 27 cases of colorectal adenomas and adjacent tissues (66). Adenoma size and disease stage were closely associated with fungal microbiota. Furthermore, a series of intestinal microbial biomarkers were identified to distinguish CRC patients from controls (7, 66). This indicates the potential of intestinal microbiome as a tool towards targeted non-invasive biomarkers for CRC.

Many studies on the intestinal microbiome of CRC are performed using fecal samples because of its easy and non-invasive procedure. However, tissue samples from colonic mucosa are more valuable to disentangle the physiopathology of CRC disease and cumulative studies have shown different microbiome profiles between mucosal and fecal samples (67–71). So far, the unified microbial community structure associated with CRC has not been determined and sample collection is another challenging in microbiome studies of CRC. Accordingly, further studies are warranted to determine which is more representative of the real microbial structure change.

### HCC

Hepatitis virus is well known to be closely related to HCC, but the role of bacteria in the occurrence and development of HCC remains unclear. In 1992, the Frederick Cancer Research Center identified a spiral bacterium-*Helicobacter hepaticus* from the liver tissue, and considered it as a cause of hepatitis and liver tumors in mice (14). Since then, scientists have carried out a series of studies on the association between *Helicobacter* spp. and chronic liver diseases (72–75). The *Helicobacter* 16S rRNA gene was sequentially detected in liver tissues of patients with chronic liver diseases and HCC (76–78). In addition, an association between H. pylori infection and mortality of patients with HCC has been observed (79). However, studies on the intratumoral microbiota of HCC are scarce and further investigations on the role of other bacteria in HCC are needed.

The above are reported researches on intratumoral microbiome of gastrointestinal cancers, and we summarize the intratumoral microbiome in **Table 1**.

**Table 1:** Gastrointestinal cancer-associated intratumoral microbiota.

Cancer type	Bacteria	References	Fungi	References
Esophageal cancer	*Escherichia coli* *Fusobacterium nucleatum* *Lactobacillus fermentum*	(22, 23) (22, 26) (24, 25)	–	–
Gastric cancer	*Helicobacter pylori* *Streptococcus* *Lactobacillus* *Escherichia-Shigella* *Lachnospiraceae*	(28, 29) (31, 32) (31, 33) (33) (31, 33)	–	–
Pancreatic cancer	*Pseudoxanthomonas* *Streptomyces* *Saccharopolyspora* *Bacillus clausii*	(11, 38) (11, 38) (11, 38) (11, 38)	*Malassezia*	(39, 40)
Colorectal cancer	*Escherichia coli* *Peptostreptococcus anaerobius* *Fusobacterium nucleatum* *Bacteroides fragilis* *Enterococcus faecalis* *Salmonella*	(55, 59) (57, 58) (51, 53, 56, 62, 64) (54, 60) (61, 63) (61, 63)	*Ascomycota* *Basidiomycota*	(7, 66) (7, 66)
Hepatocellular carcinoma	*Helicobacter hepaticum* *Helicobacter pylori*	(14, 72, 75) (73, 74, 76, 77)	*-*	–

## Crosstalk between intratumoral microbiome and TME

**Figure 1:**
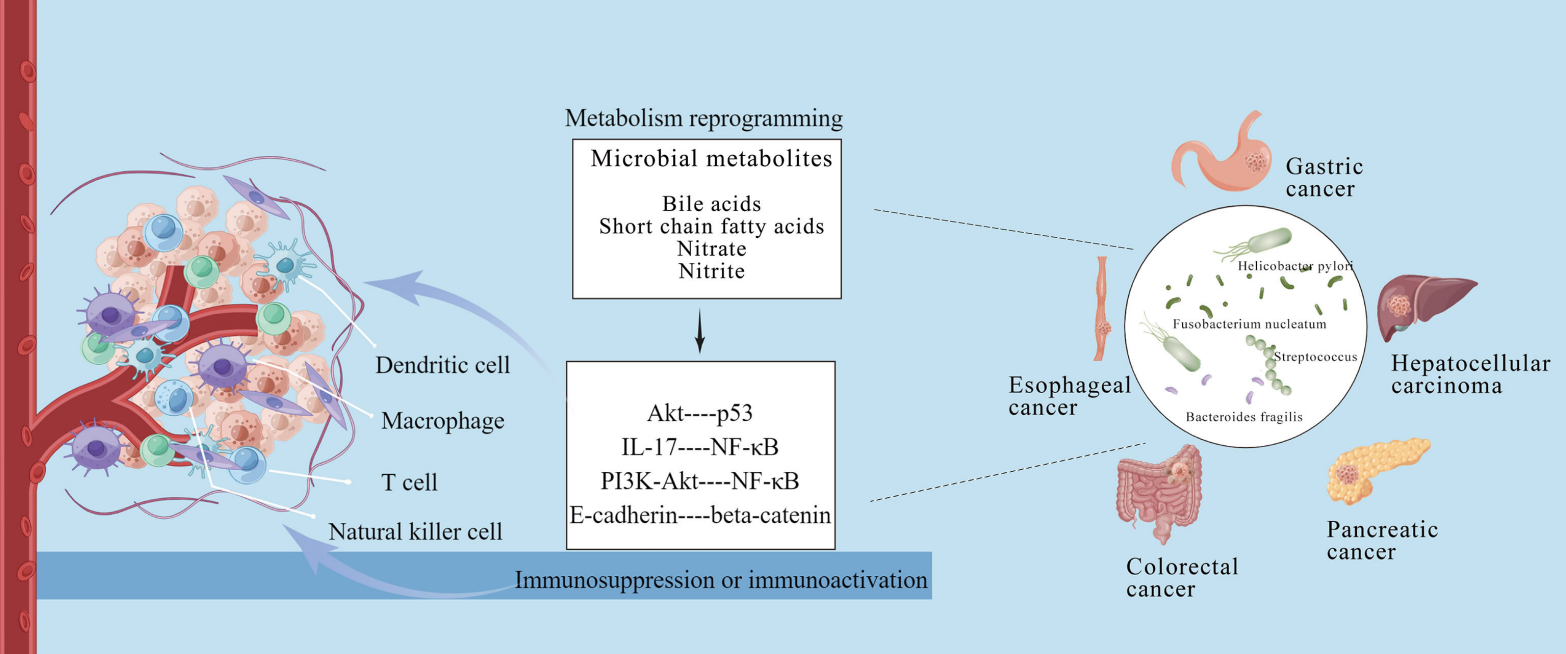
Crosstalk between intratumoral microbiome of gastrointestinal cancers and tumor microenvironment. The intratumoral microbiota can induce immunosuppression or immunoactivation *via* metabolism reprogramming, and form a heterogeneity tumor microenvironment to affect tumor occurrence and development. This figure is drawn by Figdraw.

Emerging studies have demonstrated intratumoral microbiome as a component of TME, and the crosstalk between intratumoral microbiome and TME is mutual and highly dynamic (1, 15). Specifically, the intratumoral microbiome can induce immunosuppression or immunoactivation, reprogram tumor metabolism, and form a heterogeneity TME so as to promote or inhibit tumor development (80, 81). The potential crosstalk reported between the microbiome and TME is shown in **Figure 1**.

The reprogramming of immune infiltration plays important roles in the crosstalk between the intratumoral microbiome and TME (2, 82). A recent research revealed that microbial components (e.g., DNA, RNA, bacterial peptides, and lipopolysaccharides) could be detected in tumor and immune cells, indicating that intratumoral microbiome might affect immune infiltration in TME (15). Similarly, bacterial polypeptide fragments were found to be directly presented on the surface of tumor cells or antigen-presenting cells through human leukocyte antigens. They could promote T cell activation and potential tumor immune response (83). Tumor monocytes can be induced to produce interferon-I, regulate macrophage polarization, promote communication of natural killer and dendritic cells, and reprogram the TME (84). In addition, the microbiota may regulate immune invasion in different tumors in different ways. Proteus bacteria (mainly *E. coli*) in CRC may destroy the intestinal barrier, migrate and colonize the liver through the damaged intestinal barrier, and promote immune cell recruitment in the liver (e.g., macrophages, neutrophils, and monocytes) to induce liver metastasis (85). *F. nucleatum* is reported to bind to the immunosuppressive receptor T cell immunoglobulin through its surface adhesin, thereby inhibiting T cell activation and natural killer cell lethality (86). Pushalkar et al. demonstrated that intratumoral bacteria in pancreatic cancer could promote oncogenesis by inducing innate and adaptive immune suppression (12). While bacterial ablation induced immunogenic reprogramming, including reduction of myeloid-derived suppressor cells, increase of M1 macrophage differentiation, promotion of Th1 differentiation of CD4+ T cells, and activation of CD8+ T cells (12). Mechanistically, the immune-suppression characteristic of PDAC were generated by bacteria through differentially activating select toll-like receptors in monocytic cells. Furthermore, special bacterial species were found to favor recruitment and activation of CD8+ T cells, contribute to the anti-tumor immune response and affect clinical outcomes of pancreatic cancer (11).

Many of the above findings are still arguable and need further research. While, some preclinical models have clarified potential mechanisms by which microbiota can contribute to tumorigenesis and established a more causative role. *P. gingivalis* infection was found to enhance proliferation of PDAC cells by cell lines and a xenograft model (87). This was independent of TLR2 signaling and associated with augmentation of the Akt signaling pathway (87). Similarly, Akt signaling pathway activated by *H pylori* can also result in subsequent degradation of tumor suppressor p53 in gastric epithelial cells and increase survival of gastric epithelial cells with sustained DNA damage (88). Enterotoxigenic *B. fragilis* can lead to colitis that triggers IL-17 production and inflammatory pathway activation, such as NF-κB, to facilitate tumor growth (54, 89). While PI3K-Akt pathway activated in CRC can lead to increased cell proliferation and NF-κB activation (58). *F. nucleatum* may selectively bind and activate E-cadherin/beta-catenin signaling *via* its FadA adhesin, inducing inflammation and CRC (90). *Enterococcus faecalis* in CRC induces superoxide production, thus damaging the DNA of epithelial cells (91, 92).

Additionally, tumor microbiome is reported to directly affect the response to antitumor therapies (93, 94). The enrichment of Firmicutes, reciprocal changes in abundance of Verrucomicrobia and Proteobacteria, were found to be correlated with better immunocheckpoint inhibitor (ICI) response across various tumors (93, 94). In addition, *F. nucleatum* is found to promote the resistance of colorectal cancer to chemotherapy (95). Mechanistically, *F. nucleatum* activated the autophagy pathway by targeting at TLR4 and MYD88 innate immune signaling and specific microRNAs to promote chemoresistance (95). A study about CD47-based cancer immunotherapy revealed that accumulative *Bifidobacterium* in TME facilitates local anti-CD47 treatment by a stimulator of interferon genes (STING)- and interferon-dependent fashion (96). Besides, disruption of gut microbiota is estimated to affect the response of tumors to immunotherapy and chemotherapy both in preclinical models and cancer patients (97–100). Antibiotic treatment can reduce response to ICI, while the presence of certain bacteria strains correlates with better outcomes (10, 101). Mechanistically, gut microbiota could modulate the expression of immune checkpoints, the function of dendritic cell, the homing and recruitment of lymphocyte (82, 83), as well as the production of critical metabolites, such as short chain fatty acids (SCFA) (102, 103).

The microbiome participates in the modulation of human metabolism and microbial dysbiosis can induce systemic metabolic alterations (104, 105). To date, intratumoral microbiome has been shown to reprogram tumor metabolism through derived metabolites, and a differential enrichment of metabolic functional pathways induced by microbiota may correlate with clinical outcome. As reported, patients with PDAC who are enrichment in xenobiotics biodegradation and lipids metabolism pathways have shown better outcomes (11). Metabolites, such as the secondary bile acids (e.g., deoxycholic acid, lithocholic acid) and SCFA (e.g., butyrate), have been reported to regulate inflammation and T cell differentiation (106–108) and implicated in the tumorigenesis (109, 110). HCC oncogenesis is found to be closely associated with intestinal flora, bile acid metabolism, and tumor immunity (111). Gut microbiota-mediated bile acid metabolism regulates the occurrence and progression of HCC by affecting the accumulation of hepatic CXCR6^+^ natural killer T (NKT) cells through mediating the expression of CXCL16 in liver sinusoidal endothelial cells (111). SCFAs, mainly butyrate, can directly modulate CD8(+) cytotoxic T lymphocytes and Tc17 cells, as well as induce senescence-like phenotypes and the development of CRC (106, 107). The secondary metabolites produced by microbiota, such as reactive nitrate and nitrite, can lead to accumulation of carcinogenic N-nitroso compounds and associate with tumor development (26, 112). These studies shed light on a new theoretical basis for tumor treatment by regulating microbial metabolism and the microbiota.

## Conclusions

Despite decades of research, gastrointestinal cancers remain the most lethal malignant tumor with limited treatment options and poor clinical outcomes. Emerging understanding of microbiome in gastrointestinal cancers will potentially provide new insights and opportunities for the development of novel biomarkers and treatment strategies. Current studies have revealed the role of microbiota in regulating tumorigenesis, development, and response to antitumor treatment, while challenges remain in our understanding of intratumoral microbiome. For example, how are the mechanisms of action of intratumoral microbiome in various tumor types? How are they different? What are the effects of microbial metabolites on tumors? How does intratumoral microbiota regulate the inflammatory carcinogenic pathway and immune response? Moreover, research discoveries on the various types of tumors have been discrepant, and this may be due to several factors, such as the tumor type, research methods, and microbial complexity. Moreover, almost no good clinical data exists for the claims. Therefore, the diversity, origin, and mechanism of action of intratumoral microbiome require further clarification. However, intratumoral microbiome has the potential to be used as diagnostic tools for tumor typing and to be considered as a potential new therapeutic target. Further in-depth researches on the intratumoral microbiome are expected to reveal the complex correlation between intratumoral microbiome and gastrointestinal cancers. Overall, this exciting field will allow us to explore the mystery of carcinogenesis from another perspective and to discover novel targets for precision medicine in the management of gastrointestinal cancers.

## Author contributions

SL: Data curation, Writing-original draft preparation. WL, QL: Writing- reviewing and editing. All authors contributed to the article and approved the submitted version.

## Funding

This study was supported by Key project of Science and Technology, Tianjin Municipal Science and Technology Bureau (19YFZCSY00020).

## Conflict of interest

The authors declare that the research was conducted in the absence of any commercial or financial relationships that could be construed as a potential conflict of interest.

## Publisher’s note

All claims expressed in this article are solely those of the authors and do not necessarily represent those of their affiliated organizations, or those of the publisher, the editors and the reviewers. Any product that may be evaluated in this article, or claim that may be made by its manufacturer, is not guaranteed or endorsed by the publisher.

## References

[1] Sepich-Poore GD Zitvogel L Straussman R Hasty J Wargo JA Knight R. The microbiome and human cancer. Science (2021) 371(6536):eabc4552. doi: 10.1126/science.abc4552 33766858 PMC8767999

[2] Poutahidis T. Erdman s E.Commensal bacteria modulate the tumor microenvironment. Cancer Lett (2016) 380(1):356–8. doi: 10.1016/j.canlet.2015.12.028 PMC4942371 26739062

[3] De Martel C Ferlay J Franceschi S Vignat J Bray F Forman D. Global burden of cancers attributable to infections in 2008: a review and synthetic analysis. Lancet Oncol (2012) 13(6):607–15. doi: 10.1016/S1470-2045(12)70137-7 22575588

[4] Human Microbiome Project C. Structure, function and diversity of the healthy human microbiome. Nature (2012) 486(7402):207–14. doi: 10.1038/nature11234 PMC3564958 22699609

[5] Chen D Wu J Jin D Wang B Cao H. Fecal microbiota transplantation in cancer management: Current status and perspectives. Int J Cancer (2019) 145(8):2021–31. doi: 10.1002/ijc.32003 PMC6767494 30458058

[6] Ren Z Li A Jiang J Zhou L Yu Z Lu H. Gut microbiome analysis as a tool towards targeted non-invasive biomarkers for early hepatocellular carcinoma. Gut (2019) 68(6):1014–23. doi: 10.1136/gutjnl-2017-315084 PMC6580753 30045880

[7] Coker OO Nakatsu G Dai RZ Wu WKK Wong SH Ng SC. Enteric fungal microbiota dysbiosis and ecological alterations in colorectal cancer. Gut (2019) 68(4):654–62. doi: 10.1136/gutjnl-2018-317178 PMC6580778 30472682

[8] Nakatsu G Zhou H Wu WKK Wong SH Coker OO Dai Z. Alterations in enteric virome are associated with colorectal cancer and survival outcomes. Gastroenterology (2018) 155(2):529–541.e5. doi: 10.1053/j.gastro.2018.04.018 29689266

[9] Elinav E Nowarski R Thaiss CA Hu B Jin C Flavell RA. Inflammation-induced cancer: crosstalk between tumours, immune cells and microorganisms. Nat Rev Cancer (2013) 13(11):759–71. doi: 10.1038/nrc3611 24154716

[10] Routy B Le Chatelier E Derosa L Duong CPM Alou MT Daillère R. Gut microbiome influences efficacy of PD-1-based immunotherapy against epithelial tumors. Science (2018) 359(6371):91–7. doi: 10.1126/science.aan3706 29097494

[11] Riquelme E Zhang Y Zhang L Montiel M Zoltan M Dong W. Tumor microbiome diversity and composition influence pancreatic cancer outcomes. Cell (2019) 178(4):795–806.e12. doi: 10.1016/j.cell.2019.07.008 31398337 PMC7288240

[12] Pushalkar S Hundeyin M Daley D Zambirinis CP Kurz E Mishra A. The pancreatic cancer microbiome promotes oncogenesis by induction of innate and adaptive immune suppression. Cancer Discovery (2018) 8(4):403–16. doi: 10.1158/2159-8290.CD-17-1134 PMC6225783 29567829

[13] Sepich-Poore GD Carter H. Knight R.Intratumoral bacteria generate a new class of therapeutically relevant tumor antigens in melanoma. Cancer Cell (2021) 39(5):601–3. doi: 10.1016/j.ccell.2021.04.008 33974857

[14] Fox JG Dewhirst FE Tully JG Paster BJ Yan L Taylor NS. Helicobacter hepaticus sp. nov., a microaerophilic bacterium isolated from livers and intestinal mucosal scrapings from mice. J Clin Microbiol (1994) 32(5):1238–45. doi: 10.1128/jcm.32.5.1238-1245.1994 PMC263656 8051250

[15] Nejman D Livyatan I Fuks G Gavert N Zwang Y Geller LT. The human tumor microbiome is composed of tumor type-specific intracellular bacteria. Science (2020) 368(6494):973–80. doi: 10.1126/science.aay9189 PMC7757858 32467386

[16] Ramirez-Labrada AG Isla D Artal A Arias M Rezusta A Pardo J. The influence of lung microbiota on lung carcinogenesis, immunity, and immunotherapy. Trends Cancer (2020) 6(2):86–97. doi: 10.1016/j.trecan.2019.12.007 32061309

[17] Oze I Matsuo K Wakai K Nagata C Mizoue T Tanaka K. Alcohol drinking and esophageal cancer risk: an evaluation based on a systematic review of epidemiologic evidence among the Japanese population. Jpn J Clin Oncol (2011) 41(5):677–92. doi: 10.1093/jjco/hyr026 21430021

[18] Lin Y Totsuka Y He Y Kikuchi S Qiao Y Ueda J. Epidemiology of esophageal cancer in Japan and China. J Epidemiol (2013) 23(4): 233–42. doi: 10.2188/jea.JE20120162 PMC3709543 23629646

[19] Snider EJ Compres G Freedberg DE Khiabanian H Nobel YR Stump S. Alterations to the esophageal microbiome associated with progression from barrett's esophagus to esophageal adenocarcinoma. Cancer Epidemiol Biomarkers Prev (2019) 28(10):1687–93. doi: 10.1158/1055-9965.EPI-19-0008 PMC6774849 31466948

[20] Plaza-Diaz J Alvarez-Mercado AI Ruiz-Marin CM Reina-Pérez I Pérez-Alonso AJ Sánchez-Andujar MB. Association of breast and gut microbiota dysbiosis and the risk of breast cancer: a case-control clinical study. BMC Cancer (2019) 19(1):495. doi: 10.1186/s12885-019-5660-y 31126257 PMC6534876

[21] Yang L Lu X Nossa CW Francois F Peek RM Pei Z. Inflammation and intestinal metaplasia of the distal esophagus are associated with alterations in the microbiome. Gastroenterology (2009) 137(2):588–97. doi: 10.1053/j.gastro.2009.04.046 PMC2963147 19394334

[22] Ajayi TA Cantrell S Spann A Garman KS. Barrett's esophagus and esophageal cancer: Links to microbes and the microbiome. PLos Pathog (2018) 14(12):e1007384. doi: 10.1371/journal.ppat.1007384 30571768 PMC6301555

[23] Zaidi AH Kelly LA Kreft RE Barlek M Omstead AN Matsui D. Associations of microbiota and toll-like receptor signaling pathway in esophageal adenocarcinoma. BMC Cancer (2016) 16:52. doi: 10.1186/s12885-016-2093-8 26841926 PMC4739094

[24] Elliott DRF Walker AW O'donovan M Parkhill J Fitzgerald RC. A non-endoscopic device to sample the oesophageal microbiota: a case-control study. Lancet Gastroenterol Hepatol (2017) 2(1):32–42. doi: 10.1016/S2468-1253(16)30086-3 28404012 PMC5656094

[25] Lv J Guo L Liu JJ Zhao HP Zhang J Wang JH. Alteration of the esophageal microbiota in barrett's esophagus and esophageal adenocarcinoma. World J Gastroenterol (2019) 25(18):2149–61. doi: 10.3748/wjg.v25.i18.2149 PMC6526156 31143067

[26] Yang W Chen CH Jia M Xing X Gao L Tsai HT. Tumor-associated microbiota in esophageal squamous cell carcinoma. Front Cell Dev Biol (2021) 9:641270. doi: 10.3389/fcell.2021.641270 33681225 PMC7930383

[27] Yamamura K Baba Y Nakagawa S Mima K Miyake K Nakamura K. Human microbiome fusobacterium nucleatum in esophageal cancer tissue is associated with prognosis. Clin Cancer Res (2016) 22(22):5574–81. doi: 10.1158/1078-0432.CCR-16-1786 27769987

[28] Cai H Ye F Michel A Murphy G Sasazuki S Taylor PR. Helicobacter pylori blood biomarker for gastric cancer risk in East Asia. Int J Epidemiol (2016) 45(3):774–81. doi: 10.1093/ije/dyw078 PMC5841796 27170766

[29] Sung H Ferlay J Siegel RL Laversanne M Soerjomataram I Jemal A. Global cancer statistics 2020: GLOBOCAN estimates of incidence and mortality worldwide for 36 cancers in 185 countries. CA Cancer J Clin (2021) 71(3):209–49. doi: 10.3322/caac.21660 33538338

[30] Correa P Houghton J. Carcinogenesis of helicobacter pylori. Gastroenterology (2007) 133(2):659–72. doi: 10.1053/j.gastro.2007.06.026 17681184

[31] Eun CS Kim BK Han DS Kim SY Kim KM Choi BY. Differences in gastric mucosal microbiota profiling in patients with chronic gastritis, intestinal metaplasia, and gastric cancer using pyrosequencing methods. Helicobacter (2014) 19(6):407–16. doi: 10.1111/hel.12145 25052961

[32] Dai D Yang Y Yu J Dang T Qin W Teng L. Interactions between gastric microbiota and metabolites in gastric cancer. Cell Death Dis (2021) 12(12):1104. doi: 10.1038/s41419-021-04396-y 34819503 PMC8613192

[33] Wang L Zhou J Xin Y Geng C Tian Z Yu X. Bacterial overgrowth and diversification of microbiota in gastric cancer. Eur J Gastroenterol Hepatol (2016) 28(3):261–6. doi: 10.1097/MEG.0000000000000542 PMC4739309 26657453

[34] Dicksved J Lindberg M Rosenquist M Enroth H Jansson JK Engstrand L. Molecular characterization of the stomach microbiota in patients with gastric cancer and in controls. J Med Microbiol (2009) 58(Pt 4):509–16. doi: 10.1099/jmm.0.007302-0 19273648

[35] Aviles-Jimenez F Vazquez-Jimenez F Medrano-Guzman R Mantilla A Torres J. Stomach microbiota composition varies between patients with non-atrophic gastritis and patients with intestinal type of gastric cancer. Sci Rep (2014) 4:4202. doi: 10.1038/srep04202 24569566 PMC3935187

[36] Leung A Tsoi H. Yu J.Fusobacterium and escherichia: models of colorectal cancer driven by microbiota and the utility of microbiota in colorectal cancer screening. Expert Rev Gastroenterol Hepatol (2015) 9(5):651–7. doi: 10.1586/17474124.2015.1001745 25582922

[37] Geller LT Barzily-Rokni M Danino T Jonas OH Shental N Nejman D. Potential role of intratumor bacteria in mediating tumor resistance to the chemotherapeutic drug gemcitabine. Science (2017) 357(6356):1156–60. doi: 10.1126/science.aah5043 PMC5727343 28912244

[38] Bradley CA. Tumour microbiome defines outcomes. Nat Rev Cancer (2019) 19(10):545. doi: 10.1038/s41568-019-0201-1 31471581

[39] Aykut B Pushalkar S Chen R Li Q Abengozar R Kim JI. The fungal mycobiome promotes pancreatic oncogenesis *via* activation of MBL. Nature (2019) 574(7777):264–7. doi: 10.1038/s41586-019-1608-2 PMC6858566 31578522

[40] Lee SC. mSphere of influence: the mycobiota in human health and disease. mSphere (2020) 5(1):e00974–19. doi: 10.1128/mSphere.00974-19 31969481 PMC6977182

[41] Vitiello GA Cohen DJ. Miller G.Harnessing the microbiome for pancreatic cancer immunotherapy. Trends Cancer (2019) 5(11):670–6. doi: 10.1016/j.trecan.2019.10.005 31735286

[42] Li JJ Zhu M Kashyap PC Chia N Tran NH McWilliams RR. The role of microbiome in pancreatic cancer. Cancer Metastasis Rev (2021) 40(3):777–89. doi: 10.1007/s10555-021-09982-2 PMC8402962 34455517

[43] Bray F Ferlay J Soerjomataram I Siegel RL Torre LA Jemal A. Global cancer statistics 2018: GLOBOCAN estimates of incidence and mortality worldwide for 36 cancers in 185 countries. CA Cancer J Clin (2018) 68(6):394–424. doi: 10.3322/caac.21492 30207593

[44] Louis P Hold GL. Flint h J.The gut microbiota, bacterial metabolites and colorectal cancer. Nat Rev Microbiol (2014) 12(10):661–72. doi: 10.1038/nrmicro3344 25198138

[45] Nakatsu G Li X Zhou H Sheng J Wong SH Wu William KK. Gut mucosal microbiome across stages of colorectal carcinogenesis. Nat Commun (2015) 6:8727. doi: 10.1038/ncomms9727 26515465 PMC4640069

[46] Chiu DK Tse AP Xu IM Di CJ Lai RK Li LL. Hypoxia inducible factor HIF-1 promotes myeloid-derived suppressor cells accumulation through ENTPD2/CD39L1 in hepatocellular carcinoma. Nat Commun (2017) 8(1):517. doi: 10.1038/s41467-017-00530-7 28894087 PMC5593860

[47] Coker OO Dai Z Nie Y Zhao G Cao L Nakatsu G. Mucosal microbiome dysbiosis in gastric carcinogenesis. Gut (2018) 67(6):1024–32. doi: 10.1136/gutjnl-2017-314281 PMC5969346 28765474

[48] Jobin C. Colorectal cancer: looking for answers in the microbiota. Cancer Discovery (2013) 3(4):384–7. doi: 10.1158/2159-8290.CD-13-0042 PMC3625977 23580283

[49] Tlaskalova-Hogenova H Vannucci L Klimesova K Stepankova R Krizan J Kverka M. Microbiome and colorectal carcinoma: insights from germ-free and conventional animal models. Cancer J (2014) 20(3):217–24. doi: 10.1097/PPO.0000000000000052 24855011

[50] Kostic AD Chun E Robertson L Glickman JN Gallini CA Michaud M. Fusobacterium nucleatum potentiates intestinal tumorigenesis and modulates the tumor-immune microenvironment. Cell Host Microbe (2013) 14(2):207–15. doi: 10.1016/j.chom.2013.07.007 PMC3772512 23954159

[51] Mima K Cao Y Chan AT Qian ZR Nowak JA Masugi Y. Fusobacterium nucleatum in colorectal carcinoma tissue according to tumor location. Clin Transl Gastroenterol (2016) 7(11):e200. doi: 10.1038/ctg.2016.53 27811909 PMC5543402

[52] Chen D Zhang Y Wang W Chen H Ling T Yang R. Identification and characterization of robust hepatocellular carcinoma prognostic subtypes based on an integrative metabolite-protein interaction network. Adv Sci (Weinh) (2021) 8(17):e2100311. doi: 10.1002/advs.202100311 34247449 PMC8425875

[53] Bashir A Miskeen AY Bhat A Fazili KM Ganai BA. Fusobacterium nucleatum: an emerging bug in colorectal tumorigenesis. Eur J Cancer Prev (2015) 24(5):373–85. doi: 10.1097/CEJ.0000000000000116 25569450

[54] Dejea CM Fathi P Craig JM Boleij A Taddese R Geis AL. Patients with familial adenomatous polyposis harbor colonic biofilms containing tumorigenic bacteria. Science (2018) 359(6375):592–7. doi: 10.1126/science.aah3648 PMC5881113 29420293

[55] Bonnet M Buc E Sauvanet P Darcha C Dubois D Pereira B. Colonization of the human gut by e. coli and colorectal cancer risk. Clin Cancer Res (2014) 20(4):859–67. doi: 10.1158/1078-0432.CCR-13-1343 24334760

[56] Castellarin M Warren RL Freeman JD Dreolini L Krzywinski M Strauss J. Fusobacterium nucleatum infection is prevalent in human colorectal carcinoma. Genome Res (2012) 22(2):299–306. doi: 10.1101/gr.126516.111 22009989 PMC3266037

[57] Tsoi H Chu ESH Zhang X Sheng J Nakatsu G Ng SC. Peptostreptococcus anaerobius induces intracellular cholesterol biosynthesis in colon cells to induce proliferation and causes dysplasia in mice. Gastroenterology (2017) 152(6):1419–1433.e5. doi: 10.1053/j.gastro.2017.01.009 28126350

[58] Long X Wong CC Tong L Chu ESH Ho Szeto C Go MYY. Peptostreptococcus anaerobius promotes colorectal carcinogenesis and modulates tumour immunity. Nat Microbiol (2019) 4(12):2319–30. doi: 10.1038/s41564-019-0541-3 31501538

[59] Veziant J Gagniere J Jouberton E Bonnin V Sauvanet P Pezet D. Association of colorectal cancer with pathogenic escherichia coli: Focus on mechanisms using optical imaging. World J Clin Oncol (2016) 7(3):293–301. doi: 10.5306/wjco.v7.i3.293 27298769 PMC4896897

[60] Chung L Thiele Orberg E Geis AL Chan JL Fu K DeStefano Shields CE. Bacteroides fragilis toxin coordinates a pro-carcinogenic inflammatory cascade *via* targeting of colonic epithelial cells. Cell Host Microbe (2018) 23(2):203–214.e5. doi: 10.1016/j.chom.2018.01.007 29398651 PMC5954996

[61] Li S Liu J Zheng X Ren L Yang Y Li W. Tumorigenic bacteria in colorectal cancer: mechanisms and treatments. Cancer Biol Med (2021). doi: 10.20892/j.issn.2095-3941.2020.0651 PMC8832957 34586760

[62] Kostic AD Gevers D Pedamallu CS Michaud M Duke F Earl AM. Genomic analysis identifies association of fusobacterium with colorectal carcinoma. Genome Res (2012) 22(2):292–8. doi: 10.1101/gr.126573.111 PMC3266036 22009990

[63] Henstra C Van Praagh J Olinga P Nagelkerke A. The gastrointestinal microbiota in colorectal cancer cell migration and invasion. Clin Exp Metastasis (2021) 38(6):495–510. doi: 10.1007/s10585-021-10130-x 34748126

[64] Bullman S Pedamallu CS Sicinska E Clancy TE Zhang X Cai D. Analysis of fusobacterium persistence and antibiotic response in colorectal cancer. Science (2017) 358(6369):1443–8. doi: 10.1126/science.aal5240 PMC5823247 29170280

[65] Andzinski L Kasnitz N Stahnke S Wu CF Gereke M von Köckritz-Blickwede M. Type I IFNs induce anti-tumor polarization of tumor associated neutrophils in mice and human. Int J Cancer (2016) 138(8):1982–93. doi: 10.1002/ijc.29945 26619320

[66] Luan C Xie L Yang X Miao H Lv N Zhang R. Dysbiosis of fungal microbiota in the intestinal mucosa of patients with colorectal adenomas. Sci Rep (2015) 5:7980. doi: 10.1038/srep07980 25613490 PMC4648387

[67] Yu LC Wei SC Ni YH. Impact of microbiota in colorectal carcinogenesis: lessons from experimental models. Intest Res (2018) 16(3):346–57. doi: 10.5217/ir.2018.16.3.346 PMC6077307 30090033

[68] Ahn J Sinha R Pei Z Dominianni C Wu J Shi J. Human gut microbiome and risk for colorectal cancer. J Natl Cancer Inst (2013) 105(24):1907–11. doi: 10.1093/jnci/djt300 PMC3866154 24316595

[69] Zackular JP Rogers MA Ruffin MTT Schloss PD. The human gut microbiome as a screening tool for colorectal cancer. Cancer Prev Res (Phila) (2014) 7(11):1112–21. doi: 10.1158/1940-6207.CAPR-14-0129 PMC4221363 25104642

[70] Feng Q Liang S Jia H Stadlmayr A Tang L Lan Z. Gut microbiome development along the colorectal adenoma-carcinoma sequence. Nat Commun (2015) 6:6528. doi: 10.1038/ncomms7528 25758642

[71] Wirbel J Pyl PT Kartal E Zych K Kashani A Milanese A. Meta-analysis of fecal metagenomes reveals global microbial signatures that are specific for colorectal cancer. Nat Med (2019) 25(4):679–89. doi: 10.1038/s41591-019-0406-6 PMC7984229 30936547

[72] Dore MP Realdi G Mura D Graham DY Sepulveda AR. Helicobacter infection in patients with HCV-related chronic hepatitis, cirrhosis, and hepatocellular carcinoma. Dig Dis Sci (2002) 47(7):1638–43. doi: 10.1023/A:1015848009444 12141829

[73] Pellicano R Leone N Berrutti M Cutufia MA Fiorentino M Rizzetto M. Helicobacter pylori seroprevalence in hepatitis c virus positive patients with cirrhosis. J Hepatol (2000) 33(4):648–50. doi: 10.1016/S0168-8278(00)80018-5 11059871

[74] Avenaud P Marais A Monteiro L Le Bail B Bioulac Sage P Balabaud C. Detection of helicobacter species in the liver of patients with and without primary liver carcinoma. Cancer (2000) 89(7):1431–9. doi: 10.1002/1097-0142(20001001)89:7<1431::AID-CNCR4>3.0.CO;2-5 11013355

[75] Yang J Ji S Zhang Y Wang J. Helicobacter hepaticus infection in primary hepatocellular carcinoma tissue. Singapore Med J (2013) 54(8):451–7. doi: 10.11622/smedj.2013153 24005452

[76] Nilsson HO Mulchandani R Tranberg KG Stenram U Wadström T. Helicobacter species identified in liver from patients with cholangiocarcinoma and hepatocellular carcinoma. Gastroenterology (2001) 120(1):323–4. doi: 10.1053/gast.2001.21382 11246512

[77] Fan XG Zou YY Wu AH Li TG Hu GL Zhang Z. Seroprevalence of helicobacter pylori infection in patients with hepatitis b. Br J BioMed Sci (1998) 55(3):176–8. doi: 10.1097/00022744-199809000-00014 10367401

[78] Rocha M Avenaud P Menard A Le Bail B Balabaud C Bioulac-Sage P. Association of helicobacter species with hepatitis c cirrhosis with or without hepatocellular carcinoma. Gut (2005) 54(3):396–401. doi: 10.1136/gut.2004.042168 15710989 PMC1774397

[79] Yang JD Mohamed EA Aziz AO Shousha HI Hashem MB Nabeel MM. Characteristics, management, and outcomes of patients with hepatocellular carcinoma in Africa: a multicountry observational study from the Africa liver cancer consortium. Lancet Gastroenterol Hepatol (2017) 2(2):103–11. doi: 10.1016/S2468-1253(16)30161-3 28403980

[80] Belkaid Y Hand TW. Role of the microbiota in immunity and inflammation. Cell (2014) 157(1):121–41. doi: 10.1016/j.cell.2014.03.011 PMC4056765 24679531

[81] Dzutsev A Badger JH Perez-Chanona E Roy S Salcedo R Smith CK. Microbes and cancer. Annu Rev Immunol (2017) 35:199–228. doi: 10.1146/annurev-immunol-051116-052133 28142322

[82] Erdman SE Poutahidis T. The microbiome modulates the tumor macroenvironment. Oncoimmunology (2014) 3:e28271. doi: 10.4161/onci.28271 25050199 PMC4063141

[83] Cullin N Azevedo Antunes C Straussman R Stein-Thoeringer CK Elinav E. Microbiome and cancer. Cancer Cell (2021) 39(10):1317–41. doi: 10.1016/j.ccell.2021.08.006 34506740

[84] Lam KC Araya RE Huang A Chen Q Di Modica M Rodrigues RR. Microbiota triggers STING-type I IFN-dependent monocyte reprogramming of the tumor microenvironment. Cell (2021) 184(21):5338–5356.e21. doi: 10.1016/j.cell.2021.09.019 34624222 PMC8650838

[85] Bertocchi A Carloni S Ravenda PS Bertalot G Spadoni I Lo Cascio A. Gut vascular barrier impairment leads to intestinal bacteria dissemination and colorectal cancer metastasis to liver. Cancer Cell (2021) 39(5):708–724.e11. doi: 10.1016/j.ccell.2021.03.004 33798472

[86] Gur C Ibrahim Y Isaacson B Yamin R Abed J Gamliel M. Binding of the Fap2 protein of fusobacterium nucleatum to human inhibitory receptor TIGIT protects tumors from immune cell attack. Immunity (2015) 42(2):344–55. doi: 10.1016/j.immuni.2015.01.010 PMC4361732 25680274

[87] Gnanasekaran J Binder Gallimidi A Saba E Pandi K Eli Berchoer L Hermano E. Intracellular porphyromonas gingivalis promotes the tumorigenic behavior of pancreatic carcinoma cells. Cancers (Basel) (2020) 12(8):2331. doi: 10.3390/cancers12082331 32824786 PMC7465784

[88] Wei J Nagy TA Vilgelm A Zaika E Ogden SR Romero-Gallo J. Regulation of p53 tumor suppressor by helicobacter pylori in gastric epithelial cells. Gastroenterology (2010) 139(4):1333–43. doi: 10.1053/j.gastro.2010.06.018 PMC2949494 20547161

[89] Cheng WT Kantilal HK Davamani F. The mechanism of bacteroides fragilis toxin contributes to colon cancer formation. Malays J Med Sci (2020) 27(4):9–21. doi: 10.21315/mjms2020.27.4.2 32863742 PMC7444842

[90] Rubinstein MR Wang X Liu W Hao Y Cai G Han YW. Fusobacterium nucleatum promotes colorectal carcinogenesis by modulating e-cadherin/beta-catenin signaling *via* its FadA adhesin. Cell Host Microbe (2013) 14(2):195–206. doi: 10.1016/j.chom.2013.07.012 23954158 PMC3770529

[91] Balamurugan R Rajendiran E George S Samuel GV Ramakrishna BS. Real-time polymerase chain reaction quantification of specific butyrate-producing bacteria, desulfovibrio and enterococcus faecalis in the feces of patients with colorectal cancer. J Gastroenterol Hepatol (2008) 23(8 Pt 1):1298–303. doi: 10.1111/j.1440-1746.2008.05490.x 18624900

[92] Wang X Allen TD May RJ Lightfoot S Houchen CW Huycke MM. Enterococcus faecalis induces aneuploidy and tetraploidy in colonic epithelial cells through a bystander effect. Cancer Res (2008) 68(23):9909–17. doi: 10.1158/0008-5472.CAN-08-1551 PMC2596646 19047172

[93] Gopalakrishnan V Helmink BA Spencer CN Reuben A Wargo JA. The influence of the gut microbiome on cancer, immunity, and cancer immunotherapy. Cancer Cell (2018) 33(4):570–80. doi: 10.1016/j.ccell.2018.03.015 PMC6529202 29634945

[94] Huang C Li M Liu B Zhu H Dai Q Fan X. Relating gut microbiome and its modulating factors to immunotherapy in solid tumors: A systematic review. Front Oncol (2021) 11:642110. doi: 10.3389/fonc.2021.642110 33816289 PMC8012896

[95] Yu T Guo F Yu Y Sun T Ma D Han J. Fusobacterium nucleatum promotes chemoresistance to colorectal cancer by modulating autophagy. Cell (2017) 170(3):548–563.e16. doi: 10.1016/j.cell.2017.07.008 28753429 PMC5767127

[96] Shi Y Zheng W Yang K Harris KG Ni K Xue L. Intratumoral accumulation of gut microbiota facilitates CD47-based immunotherapy *via* STING signaling. J Exp Med (2020) 217(5):e20192282. doi: 10.1084/jem.20192282 32142585 PMC7201921

[97] Iida N Dzutsev A Stewart CA Smith L Bouladoux N Weingarten RA. Commensal bacteria control cancer response to therapy by modulating the tumor microenvironment. Science (2013) 342(6161):967–70. doi: 10.1126/science.1240527 PMC6709532 24264989

[98] Sivan A Corrales L Hubert N Williams JB Aquino-Michaels K Earley ZM. Commensal bifidobacterium promotes antitumor immunity and facilitates anti-PD-L1 efficacy. Science (2015) 350(6264): 1084–9. doi: 10.1126/science.aac4255 PMC4873287 26541606

[99] Vetizou M Pitt JM Daillere R Lepage P Waldschmitt N Flament C. Anticancer immunotherapy by CTLA-4 blockade relies on the gut microbiota. Science (2015) 350(6264):1079–84. doi: 10.1126/science.aad1329 PMC4721659 26541610

[100] Gopalakrishnan V Spencer CN Nezi L Reuben A Andrews MC Karpinets TV. Gut microbiome modulates response to anti-PD-1 immunotherapy in melanoma patients. Science (2018) 359(6371):97–103. doi: 10.1126/science.aan4236 29097493 PMC5827966

[101] Matson V Fessler J Bao R Chongsuwat T Zha Y Alegre ML. The commensal microbiome is associated with anti-PD-1 efficacy in metastatic melanoma patients. Science (2018) 359(6371):104–8. doi: 10.1126/science.aao3290 PMC6707353 29302014

[102] Yi M Yu S Qin S Liu Q Xu H Zhao W. Gut microbiome modulates efficacy of immune checkpoint inhibitors. J Hematol Oncol (2018) 11(1):47. doi: 10.1186/s13045-018-0592-6 29580257 PMC5870075

[103] Yi M Jiao D Qin S Chu Q Li A Wu K. Manipulating gut microbiota composition to enhance the therapeutic effect of cancer immunotherapy. Integr Cancer Ther (2019) 18:1–13. doi: 10.1177/1534735419876351 PMC7242797 31517538

[104] Tremaroli V. Backhed F.Functional interactions between the gut microbiota and host metabolism. Nature (2012) 489(7415):242–9. doi: 10.1038/nature11552 22972297

[105] Nicholson JK Holmes E Kinross J Burcelin R Gibson G Jia W. Host-gut microbiota metabolic interactions. Science (2012) 336(6086):1262–7. doi: 10.1126/science.1223813 22674330

[106] Arpaia N Campbell C Fan X Dikiy S van der Veeken J deRoos P. Metabolites produced by commensal bacteria promote peripheral regulatory T-cell generation. Nature (2013) 504(7480):451–5. doi: 10.1038/nature12726 PMC3869884 24226773

[107] Luu M Weigand K Wedi F Breidenbend C Leister H Pautz S. Regulation of the effector function of CD8(+) T cells by gut microbiota-derived metabolite butyrate. Sci Rep (2018) 8(1):14430. doi: 10.1038/s41598-018-32860-x 30258117 PMC6158259

[108] Smith PM Howitt MR Panikov N Michaud M Gallini CA Bohlooly-Y M. The microbial metabolites, short-chain fatty acids, regulate colonic treg cell homeostasis. Science (2013) 341(6145):569–73. doi: 10.1126/science.1241165 PMC3807819 23828891

[109] Rossi T Vergara D Fanini F Maffia M Bravaccini S Pirini F. Microbiota-Derived Metabolites Tumor Progression and Metastasis. Int J Mol Sci (2020) 21(16). doi: 10.3390/ijms21165786 PMC7460823 32806665

[110] Okumura S Konishi Y Narukawa M Sugiura Y Yoshimoto S Arai Y. Gut bacteria identified in colorectal cancer patients promote tumourigenesis *via* butyrate secretion. Nat Commun (2021) 12(1):5674. doi: 10.1038/s41467-021-25965-x 34584098 PMC8479117

[111] Jia B. Commentary: Gut microbiome-mediated bile acid metabolism regulates liver cancer *via* NKT cells. Front Immunol (2019) 10:282. doi: 10.3389/fimmu.2019.00282 30842777 PMC6391577

[112] Forsythe SJ Cole JA. Nitrite accumulation during anaerobic nitrate reduction by binary suspensions of bacteria isolated from the achlorhydric stomach. J Gen Microbiol (1987) 133(7):1845–9. doi: 10.1099/00221287-133-7-1845 3117970

